# Analecta

**Published:** 1842-07-09

**Authors:** 


### ANALECTA.

*A Cure for Sore Nipple.*—The following extract from a clinical lecture of M. Velpeau exhibits a pretty accurate view of the ordinary treatment of sore nipple.

"*Excoriations.*—Many women who suckle for the first time during the early weeks, suffer from a kind of softening, and more or less sensibility of the nipple. In many cases the nipple becomes excoriated or inflamed, or several small ulcers may form around its base. This state occurs particularly in young women, of fine, delicate skin, and lymphatic, nervous constitution; too frequent suckling, want of cleanliness, and imperfect formation of the nipple, are predisposing causes; it is easily recognised by the pain produced during the act of suckling, the red, granular, or fungous appearance of the nipple, and by a slight exudation of blood which takes place when the infant draws the breast strongly. The exciting cause of this affection is the act of suckling; the child should, therefore, be applied to the breast at certain intervals only: as a lotion, I have used a solution of salt, wine, or even brandy, with the best effects. Should these means fail, you may apply, frequently through the day, Goulard's lotion, equal parts of oil and red wine, or, if a stronger astringent seem requisite, of oil and lime water. Sir Astley Cooper spoke favourably of a lotion composed of a drachm of borax, with half an ounce of alcohol, in three ounces of water.

"When mild means, such as those I have just mentioned, fail, I have recourse to the lunar caustic or zinc lotions; the white precipitate ointment may also be employed with advantage. It is necessary, of course, to prevent any powerful substance from passing with the milk into the child's mouth; an artificial nipple must be employed. In this way excoriations of the nipple may generally be healed.

"*Fissure of the Nipple.*—The excoriations just spoken of sometimes give rise to fissure of the nipple or areola. These are sometimes very deep, give rise to considerable hæmorrhage, and occasion excessive pain. They likewise disturb the secretion of milk, and may render it impossible for the woman to continue suckling. The treatment of this unpleasant affection is the same as that already pointed out for excoriations; but it now and then becomes so insupportable, that the medical man has recourse to much more active means. Lotions, with corrosive sublimate or calomel suspended in an infusion of marshmallows, have been tried. I have found the latter beneficial, but would never think of employing so dangerous a remedy as the former. In obstinate cases you must touch the fissures with a stick of lunar caustic. The use of artificial nipples is more requisite here than in the case of simple excoriation."

A correspondent of the London Lancet, April 30th, adds:—"In a case which I attended some time ago, I tried several of the means mentioned without any effect. They are generally greasy, nasty, painful, or poisonous applications. Now, you want an application that will not be injurious to the child, and that will thicken and toughen the nipple and the surrounding integuments. It occurred to me that a solution containing tannin might have this effect. I first tried the decoction of oak bark: upon another occasion I applied the tincture of catechu. This answered perfectly: the nipple, which had been intolerably painful for weeks, and was denuded, returned to its natural state within a day or two, and the mother, who was about to wean her child in despair, was able to suckle it for more than twelve months without any inconvenience.

The tincture of catechu should be applied twice a-day with a camel's-hair pencil.

---

[The following article on copaiva has been going the rounds of the English Journals. We extract it for the same reasons: copaiva is always a disgusting, but often a most useful remedy.]

*Administration of Copaiva.*—Copaiva is most generally administered by the mouth, but sometimes in the way of clyster. It is often given in too small doses. They ought not to be less than half a drachm; and an entire drachm, given twice or thrice a day, is the most appropriate quantity. As it is a nauseous medicine, on account of the quality, permanence, and adhesiveness of its taste, various devices have been contrived for facilitating its administration. Some take it simply in water, which is stirred briskly so as to collect the copaiva in a globule in the centre. A better plan is to make it into an emulsion. For this purpose each drachm may be triturated with the yoke of one egg, to which are afterwards to be added half an ounce of aromatic, such as peppermint or cinnamon water, and then as much simple water as pleases the patient; or the copaiva may be dissolved in its own volume of spirit of nitrous ether, and then agitated with twice as much mucilage and four times as much water. A favourite method of giving copaiva in recent times is in the form of boluses, made by inclosing the drug in thin capsules of gelatin, which are dissolved in the stomach. This ingenious plan was contrived a few years ago in Paris by M. Mothes, and the process for making the capsules has been kept secret. They may be made in the following manner: the body of the capsule is formed by rounding very smoothly the end of a cylinder of iron or hard wood, four lines in diameter and a few inches in length, dipping half an inch of this end into a saturated alcoholic solution of soap kept warm; then dipping it, when the layer of soap has concreted, into a strong hot solution of gelatin once, or oftener, according to the thickness desired; and, lastly, removing the capsule by a screwing motion when the gelatin is quite dry. The top is made in the same way, but shorter and a trifle wider; and when the body is filled, and the top slipped over it, they are united by rubbing over the line of junction a camel's-hair brush moistened with hot water (Feder, in Buckner's Repertorium.) The form of pill, which, however, is ineligible on account of its insolubility, is best attained by sprinkling one part of calcined magnesia into sixteen parts of copaiva in a flat plate, and letting the compound stand till it thickens sufficiently to be worked into the proper shape. For some time past it has become fashionable in Britain to use what are called specific solutions of copaiva; for which every druggist has his formula, and which have the advantage of presenting the drug in a state of solution, and capable of being diluted without being decomposed. They are commonly made with solution of potash and spirit of nitrous ether, and the following is a convenient formula in use in this city. Boil gently for fifteen minutes two ounces of copaiva with two ounces and a half of aqua potassæ; add, when nearly cool, an ounce of spirit of nitrous ether; and, when the mixture has been at rest for twelve hours, remove the intermediate liquid from the soapy sediment which falls, and the lighter fluid which floats on the surface. In these preparations a part of the volatile oil seems to be separated, and most of the resin deposited in the form of soap. M. Velpeau, not long ago, proposed to administer copaiva in the way of clyster instead of by the mouth. He found it very efficacious when given in divided doses to the extent of an ounce daily in the form of emulsion, to which a little laudanum was added to prevent its too speedy discharge from the gut. Many now prefer the pure volatile oil to any form for administering the crude drug; and though some call in question its superiority, and a few even doubt its efficacy altogether, I am satisfied from observation, as well as many reports from medical friends, that it is at least as effectual as copaiva, efficacious in less doses, and not so apt to occasion sickness. It is best given in emulsion, composed of equal parts of the oil of rectified spirit, peppermint or cinnamon water, and syrup of mucilage. Among the inconveniences attending the use of copaiva, sickening and vomiting are the most frequent. This effect may sometimes be prevented by multiplying, but diminishing, the doses; by uniting an aromatic water with it, or by directing the patient to chew a piece of cinnamon or nutmeg after each dose. Occasionally a sharp febrile attack is occasioned when the medicine has been taken for some days in gonorrhœa; but as this attack goes off with perspiration in twenty-four or thirty-six hours, and is commonly attended with arrestment of the discharge, it ought not to occasion annoyance, and scarcely requires any treatment. The doses of the preparations of copaivæ are, Copaviæ, m. xv. ad fl. scr. iv. Copaivæ Oleum, E. m. ad m. xxx.—*Christison's Dispensatory.*

---

*Hæmorrhage from Polypus Uteri.*—Cases occasionally occur in which the existence of polypus uteri is not recognised, and the hæmorrhage which follows being frequently repeated, exhausts and wears out the patient. Local and general astringents are unavailing. If, at this period of the case, its nature is better understood, and the presence of the polypus is discovered, it becomes a question whether it should be removed. M. Lisfranc says, that the greater number of polypi of the uterus are white and fibrous, containing a very small quantity of blood vessels in their interior, but they are covered with a species of mucous membrane, forming a complete vascular net-work, whence, according to him, proceeds the hæmorrhage. If the polypus is very low down, he seizes this membrane with the thumb and index, pinches and tears it, and, in fact, completely skins the polypus. If situated too high up, he introduces the largest speculum possible, draws it downwards with hooks, sponges it, injects cold or aluminous water, and then cauterises the whole extent of the polypus with the proto-nitrate acid of mercury. In either case the hæmorrhage is at once stopped. By these means the loss of blood is arrested, and the patient enabled to get strength to undergo the necessary operation afterwards.—*Prov. Med. Jour.*, May 28, 1842, from *Bul. Gen. de Ther.*

---

*Trichiasis.*—M. Alessi considers there are three different varieties of trichiasis, dependent on peculiar causes, each variety requiring special treatment. The first is owing to a relaxation of the skin of the eyelids, and is most frequently the result of chronic ophthalmia. To remedy this he recommends cauterising, according to Heling's plan. The second variety of inversion of the eye-lashes is caused by a deviation of the bulbs, the result of superficial abscesses or pustules formed along the edge of the tarsus, the cicatrising of which has effected a change in the situation of the bulbs. M. Alessi in this case advises the excision of the edge of the eyelids, together with the bulb, taking care, in case both eyelids are affected, that the operation be not performed on both at once, lest a troublesome ankylo-blepharon be the result. The third cause is a shortening of the tarsal cartilage, following its softening from suppuration of the meibomian glands. For the cure of this variety, M. Alessi recommends an operation somewhat similar to that for ptosis—to wit, the dissection of a portion of the skin of the palpebra, sufficient, when the tarsus is brought in contact with the edge of the incised integuments, to cause eversion of the tarsus, in addition to which he incises the conjunctiva from within outwards, so as to separate its connection with the cartilage, and then brings the lips of the external wound together with sutures.—*Ibid*, from *Il Filiatre Sebezio.*

*Ipecacuanha as a Counter-Irritant.* By A. Turnbull, M. D.—I am anxious to bring under the notice of the profession a medicinal agent, which I have no doubt in the hands of judicious practitioners will prove serviceable when circumstances indicate the propriety of its use. I refer to the action of ipecacuanha and its alkaloid emetin as a counter-irritant. I am not aware that this medical substance has ever been used for the purpose of exciting counter-irritation on the surface of the body. The formulæ I find preferable are as follows:—

℞. Ipecacuanha powder, ʒij; Olive oil, ʒij; Lard, ʒiv. Or,

℞. Emetine, gr. xv; Spirit of wine, gr. xv; Lard, ʒiv.

I have not found the emetin ointment to possess any advantages over the ipecacuanha. Either of these preparations, by being rubbed on any part of the cuticular surface for a few minutes once or twice a day, produces a very numerous crop of small eruptions without any pain, which will continue out for many days. This counter-irritant is superior to the tartar-emetic ointment, as it never leaves any scars upon the skin. This is one of its peculiar advantages, when it is necessary to apply a counter-irritant to the face or neck. The pustules with some individuals assume the appearance of tetter, and the eruption is accompanied with a sensation of heat and itching. The tartar emetic appears to have a more powerful action than the ipecacuanha or emetin upon the true skin; the pustules produced by it have inflamed bases.

I have found the above formulæ of great advantage in chronic diseases of the chest and abdomen where counter-irritation is indicated. Under such circumstances, I direct one part of the chest or abdomen to be rubbed until an eruption takes place: after an interval of a day or two the ointment is applied to another part, and thus an irritation is kept up by a succession of applications to different parts of the chest. In my opinion this counter-irritant has a specific influence upon the mucous membranes of the body in the removal of disease, independently of the counter-irritation which is induced, for I have often perceived much relief effected in pulmonary affections previous to the pustules being developed. I have often witnessed the most marked beneficial effects follow the applications of the ointment over the region of the heart; the circulation becomes lowered, and nervous palpitations removed without any other remedy being used in conjunction with it.

When rubbed freely over the abdomen, I have never observed the ointment induce the slightest degree of nausea or vomiting. In cases of paralysis the parts affected ought to be well rubbed with the preparation, and the irritation should be kept up for a length of time; the peculiar irritation produced by so doing renders this remedy very efficient in the restoration of nervous energy to the affected part. The affections in which the ipecacuanha and its alkaloid will be found useful are too numerous here to specify.

When the pustules are well developed, and the patient perseveres in rubbing the ointment over them, an extreme heat with intense itching will be felt; but, unlike tartar-emetic ointment, the eruption is not disposed to assume an ulcerative character. I think this fact will establish the mildness of ipecacuanha to the tartrate of antimony as an emetic.—*London Lancet, May* 7, 1842.

*Case in which Pregnancy was unattended with the usual Signs, and in which Parturition occurred without Labour-Pains : Rupture of the Funis, which remained untied Forty-five Minutes.* By Thomas Lewis, Esq., Liverpool. Communicated by Dr. C. J. B. Williams.—The case was that of a lady, aged 31, who had noticed an enlargement of the abdomen for six or seven months. She felt certain she was not pregnant, because she had not experienced symptoms similar to those of her first pregnancy. Catamenia appeared last eight or nine months ago. External examination not proving satisfactory, examination *per vaginam* was made, which disclosed the nature of the case. The os uteri was dilated to the size of a shilling, the neck entirely expanded, and the membranes and child's head could be felt. Though informed she was pregnant she was sceptical, and made no preparation for the event. On the 5th of January the author was sent for, and found the child born before his arrival. The funis was ruptured about four inches from the umbilicus. It appears the lady had suffered from diarrhœa for two days previous. At one o'clock in the morning she awoke with, she says, griping pains in the belly. These continued until six o'clock, when she got out of bed for ease. She walked into an adjoining room, and bending herself rested her hands on a table. Suddenly the waters broke, and the child was expelled, and fell on the floor. She states positively she had no pains in the loins nor bearing-down pain previous to the expulsion of the child.

The author considers the following facts established by the case :

1. That pregnancy may occur and nearly reach its termination without many of the ordinary signs.

2. That the uterus may contract, like other hollow muscular organs, without the consciousness of the mother.

3. That rupture of the funis is attended with little or no bleeding.

The practical doctrine he infers from it is, that in cases of illegitimate births occurring suddenly, and where the child is found dead, the circumstances should be of a very decided character before the guilt of infanticide be fixed on the mother

Dr. Merriman saw nothing in the case related so very extraordinary. With regard to there being no hæmorrhage from the funis, it was well known that when this was broken by violence there was no bleeding. When torn asunder forcibly no ligature was usually necessary. He saw nothing wonderful in a patient not knowing that she was pregnant. He had seen many such cases. He was once asked to see the wife of a physician, who was stated to be labouring under ovarian dropsy, but who he found to be pregnant. On informing her of her condition, she said it was impossible. "Why so?" he inquired. "Ask my husband," was the reply of the lady.

Dr. Seymour related the case of a lady who had been married sixteen or eighteen years without being pregnant, but who at the end of that period miscarried at the fourth month, in consequence of taking medicines for removing a fancied collection of wind in the abdomen.

Dr. Johnson observed that where one woman was pregnant and denied it, twenty imagined they were so when they were not. Joanna Southcote to wit!—*London Lancet, May* 21, 1842.

This is by no means a very uncommon case, but they are often forgotten in medical testimony.

*Galvanic Forceps.*—These forceps were made by Gorck, the instrument-maker, by order of Dr. Kilian, only to see what might be their effect upon the the uterus. The blades are made of copper and zinc, and the metals are properly isolated from the hand of the accoucheur. The first experiment with the galvanic forceps was made upon a woman aged 27, of dry constitution, choleric temperament, and jaundiced complexion. The application of the forceps was decidedly indicated in this case. The head of the child, which was in the first position, remained fixed at the lower aperture of the pelvis; and the torpidity of the uterus was so great, that the child had not moved for two hours and a half; while the infiltration of the scalp was of the size of a man's fist. Before applying the forceps, Dr. Kilian had the patient bled to fourteen ounces; but this had no influence on the action of the uterus. The blades were easily introduced into the uterus; but the moment they were joined, the woman had a fresh pain, which was very violent, without being unbearable. At the same time a movement was felt in the whole uterus, which became as hard as a stone, and lost the morbid sensibility which it had shown before on each examination.

This state of things continued from the beginning to the end of the application of the forceps, and in spite of the hardness of the uterus the pains had no expulsive power. Nothing, however, indicated any spasm of the internal sexual system. After four actions with the forceps, the head cleared the lower aperture of the pelvis, and then (as well as before,) the femoral muscles underwent a spasm and trembling of an unprecedented kind. Dr. Kilian then removed his hands from the instrument, to see if the uterus, which was still contracted, would not complete the expulsion of the child's head; but this was not the case, so that he was obliged to continue the use of the forceps.

The infant immediately breathed, which was surprising, when we consider how long it had been fixed in the lower aperture of the pelvis. Hardly were the shoulders free, when the child, which was very strong, began to cry, and the pulsations of the cord immediately ceased. The uterus then contracted, and in five minutes the placenta was in the vagina. There were no pains after delivery, and the lying-in was quite regular.—*Lond. Med. Gaz.*, May 13, 1842, from *Annales de Gand*, and *Gazette des Hopitaux.*

[This is certainly a new application of galvanism.]

---

*Stricture of the Urethra, and Hypertrophy of the Bladder.* By M. Cruveilhier.—Stricture of the urethra is the most common cause of retention of urine, and of a majority of the diseases to which the urinary organs are subject.

In my opinion, modern writers have gratuitously multiplied the number of organic changes which occur in strictures of the urethra. For my part I have never met with more than one species of stricture—viz., the fibrous, or, to speak more correctly, the fibrous degeneration of the walls of the urethra, occupying either a single point of the canal all round, and thus forming the ring-structure, or extending along the canal to a length of four, six, eight, twelve lines, or more.

Besides, this distinction of strictures, according to their extent, we may admit superficial strictures, which are confined to the mucous membrane, and deep-seated strictures, in which the whole thickness of the urethra is changed into fibrous tissue. With respect to seat, the stricture may occupy any point between the orifice of the urethra and the prostatic portion, but it is almost always confined to the membranous or bulbous portions of the canal. I have never seen a stricture in the prostatic portion of the urethra. The following description of the appearances of different strictures is taken from dissections which were made with the utmost care:—

*Stricture—False Passage through the Corpus Cavernosum—Gangrene—Death.*—On opening the urethra, a false passage was observed; the instrument had traversed the upper wall of the canal, passed through the corpus cavernosum, and again entered the urethra. The stricture was four or five lines long; it was dense, fibrous, and the change of structure occupied the whole thickness of the canal; some pus was infiltrated throughout the corpus cavernosum, and the cellular tissue of the perineum was the seat of a gangrenous abscess; a portion of the lung was in a state of gray hepatisation.

Perhaps in this case the inflammation of the lung depended on the existence of pus in the corpus cavernosum. In analogous cases I have found numerous abscesses in the liver and spleen. Pus in the corpus cavernosum is placed under the same circumstances as if it were in the veins, and unless it is immediately circumscribed by adhesive inflammation, may pass into the circulation, and produce all the effects of purulent infection.

*Incontinence of Urine from Diseased Prostate—Hypertrophy of the Bladder.*—An old man, 71 years of age, was admitted into Bicetre, on the 20th of October, 1834, with retention of urine; the latter had been preceded by habitual incontinence, but the patient could give no information on the origin of his disease. A catheter was easily passed into the bladder, and allowed to remain there for fifteen days. It was then withdrawn, because the urine escaped between the instrument and the urethra; the incontinence returned.

The man now fell into a dangerous state; he was sounded every morning, and a small quantity of purulent and excessively fetid urine was drawn off; and although introduced with the greatest care, the end of the catheter was always tinged with blood, and of a very black colour when withdrawn. On the 10th of November an urinary abscess formed in front of the scrotum, and the man died on the 14th.

*Post-mortem.*—The bladder contained a small quantity of purulent urine, and a mulberry calculus as large as a walnut. The walls of the bladder were considerably hypertrophied; this condition, however, was confined to the muscular tissue, and the fatty layer between the latter and the peritoneum. The whole of the inner surface of the bladder was lined with an irregular false membrane. The prostate completely embraced the urethra; around the lower half it was very imperfectly developed, but at the upper was extremely thick and solid. The substance of the kidneys was healthy, but the cavities of their pelvis were livid, and lined with pus; the vesiculæ seminales, also, were distended with pus, and at the lower part of the urethra, in front of the scrotum, there was a large opening.

*Remarks.*—From the hypertrophy of the bladder in this case, together with its size, I am inclined to think that the incontinence of urine under which the patient laboured so long was a retention with regurgitation. The hypertrophy of the subperitoneal fatty layer would lead to the idea that the bladder remained for a great length of time inactive, and merely allowed the urine to escape as it received it. The coincidence of urinary calculus with disease of the prostate has been frequently observed, and should not be overlooked in the treatment of the latter. We should also remember that the existence of the stone may be overlooked in cases of this kind, from the difficulty of exploring the floor of the bladder; still we can make the proper examination by employing the sounds proposed by MM. Leroy or Mercier for this purpose.

The lobular arrangement of the lower portion of the prostate explains the retention of urine, and also the incontinence, when the lobes which projected into the neck of the bladder were separated from each other, as the organ became distended; they also explain why it was easy to introduce a catheter, which passed along the grooves between the prostatic lobes. The lower half of the prostate, then, was in this case made up of a number of distinct lobes, which projected separately into the bladder. As to the thick portion of prostate which embraced the upper part of the urethra, this has been occasionally seen.

In this and several other cases, I have been able to ascertain that the prostate is contained between two layers of muscle sent off from the bladder; one external and very thick; the other internal, thin, and placed between the mucous membrane and tissue of the prostate; and finally, that the prostate is traversed by a number of muscular fibres sent to it from the bladder. The thickness of the external prostatic layer, and the thinness of the internal one, explain why, in cases of hypertrophy of the prostate, its lobes always project into the bladder, and never externally to it.

The destruction of the walls of the urethra, in this case, and the infiltration of urine which ensued, were occasioned by the permanently keeping a catheter in the bladder. It is worthy of mention that I have never yet seen partial or complete hypertrophy of the prostate coincide with stricture of the urethra; nay, more, stricture of the membranous portion of the canal is almost always attended by more or less complete atrophy of the prostate gland, which often depends on chronic inflammation of the part. The two following facts support this idea:—

*Fibrous Stricture of the Urethra—Atrophy of the Prostate.*—I found, in a dead body, a stricture of the urethra occupying the bulbous and adjoining membranous part of the canal; it was fibrous, and from eight to nine lines in length. At the centre of the stricture the fibrous degeneration comprised the mucous membrane and the spongy tissue of the urethra; but towards the extremities it was confined to the mucous membrane alone. The stricture, with the neighbouring portions of the urethra, thus resembled two cones applied to each other. The urethra, between the stricture and bladder, was remarkably dilated; the verumontanum was much developed, and connected to the neck of the bladder by several bands. On pressing the prostatic portion of the urethra a small quantity of purulent matter escaped through the orifices in the verumontanum and its neighbourhood. The prostate gland, which appeared to be large, was converted into an abscess, divided into two parts by an imperfect septum; the abscess was traversed by numerous filaments, probably the vessels and excretory ducts. The bladder was small and greatly hypertrophied; it was from from four to five lines in thickness, and divided, by muscular bands, into pouches, but did not contain any calculi.

*Prostate converted into a Cyst containing a Calculus.*—A patient was admitted into hospital for a retention of urine, which he said had existed for several years. It was impossible to pass a catheter. The bladder could not be felt above the pubis, and the operation of puncture was delayed. The patient soon died.

*Post-mortem.*—Bladder very thick; gangrene extending through the mucous and muscular coats, at the posterior part; a white calculus projected into the urethra, immediately in front of the neck of the bladder; the stone occupied the site of the prostrate gland, which had been converted into a mere cyst. In the membranous portion of the canal there was a dense, circular stricture; the ureters were dilated and flexuous; the pelvis and calices of the kidneys were also much distended, but the substance of the kidneys was atrophied. The body of the right testicle contained several tubercles, and the epididymis was infiltrated with tubercular matter.

Here the various diseases of the genito-urinary system were probably consequent on the stricture of the urethra. Had the finger been passed into the rectum, the tumour would have been mistaken for an enlarged prostate; had it been known to be a calculus, nothing would have been more easy than to extract it through the rectum.

*Stricture—Fistula—Calculi.*—M. Thomas presented to the Anatomical Society the urinary organs of a man who had laboured under retention of urine for the last twenty years, and who passed his urine pretty freely by introducing a large bougie as far as the middle of the urethra. The man had no children, and having heard that this probably depended on his stricture, he resolved on going into hospital. It was impossible to pass a common sound into the bladder; a conical one was, therefore, introduced, and it was thought that it passed into the bladder, but nothing but blood came away. The man was seized next day with œdema at the glottis, and died on the following day.

The anterior half of the urethra was healthy; from its middle point there passed two canals; one, which seemed to lead to the bladder, was gangrenous, and contained one large and several small calculi; the other was surrounded by dense, fibrous tissue, and led to a large cavity in the prostate gland, filled with calculi. The prostatic pouch communicated with the bladder, and was divided into compartments by an imperfect septum.

*Remarks.*—The *fibrous* nature of stricture of the urethra seems to me to be clearly demonstrated, for I repeat that I have never seen any other form of the disease. At the strictured point the mucous membrane of the urethra completely disappears, and the spongy tissue more or less so. If we endeavour to discover the cause of this fibrous degeneration, we shall perceive that it may be explained, first, by chronic inflammation of the mucous membrane; second, by ulceration. We possess, however, too limited a number of facts relative to the state of the urethra in gonorrhœa to determine the question; for my part, I am inclined to think that strictures are the result of ulceration; for, if we admit inflammation as the cause, it is difficult to conceive how its effects should be limited to a single point of the urethra.

The therapeutic inductions derived from a knowledge of the fibrous nature of stricture are perfectly in accordance with facts. The inconveniences of forced catheterism, and of conical sounds, the superiority of dilatation over cauterisation, the necessity of continuing dilatation for a considerable length of time, the tendency of stricture to return, and the absolute necessity of having recourse, from time to time, to some means of dilatation—these are the therapeutical inductions furnished by the pathological anatomy of stricture.

It is as difficult a matter to re-establish a natural canal, when changed into fibrous tissue, as it is to make an artificial canal. Morbid fibrous tissue has a tendency to become thick and dense under the least irritation.—*Prov. Med. Journ.*, from *Annales de Chir. Franc.* February, 1842

